# Fiscal Reform in Costa Rica: Price Elasticities of Major Food Categories to Inform Decision-Making

**DOI:** 10.3389/fnut.2022.836501

**Published:** 2022-04-27

**Authors:** Eléonore Dal, Rodrigo Rivera, Cristian Morales Opazo, Mariela Madrigal

**Affiliations:** ^1^Economist, Agrifood Economics Division, FAO, Santiago, Chile; ^2^Senior Economist, Agrifood Economics Division, FAO, Santiago, Chile; ^3^Economist, FAOCR, FAO, Santiago, Chile

**Keywords:** value added tax, basic tax basket, price elasticity, nutrition, Costa Rica

## Abstract

In the context of fiscal reform in Costa Rica (value added tax revision), the definition of a new basic tax basket, “*canasta básica tributaria*” (CBT) in Spanish, incorporating nutritional criteria is underway in the country. In this study, price elasticities of major food categories were analyzed using a Quadratic Almost Ideal Demand System (QUAIDS) model. Data from the 2018 National Survey of Household Income and Expenditures was used. Measuring price elasticities is essential because it allows: knowing the extent to which food demand reacts to price changes, anticipating changes in the quantities demanded as a result of fiscal policy changes, measuring potential substitution and complementary effects between food groups, and potential nutritional effects of fiscal policies. As a result, it helps providing recommendations on the content of a CBT with nutritional criteria. Results show that the food categories with the most elastic demand are “Bread and cereals,” soft drinks and “Milk, cheese and eggs.” Substitution effects exist between the following groups: “Fruits” and “Oils and fats,” “Fruits” and “Bread and cereals,” and between “Milk, cheese and eggs” and “Oils and fats.” For this last food category and for the one which includes sweets and chocolates, the consumption decreases when the price of “Bread and cereals” increases. They are complementary goods. These relations between food groups need to be taken into account when defining a national CBT with nutritional criteria and with the objective of promoting the consumption of healthier food groups while disincentivizing the consumption of the unhealthy ones. Lastly, it is important that the consumption of the healthiest foods within each food group be fiscally promoted.

**Clinical Trial Registration:**

JEL codes: D12, H3, I18.

## Introduction

Costa Rica does not escape the double burden of hunger and malnutrition (overweight and obesity) in Latin America. Although the country has been successful in the fight against malnutrition, 5.4% of the population remains food insecure (1). Extreme poverty is stagnant at 5.8% of the population, and poverty at 21% for more than 15 years (2). At the same time, according to the 2009 National Nutrition Survey, 64.5% of the adult population is overweight or obese; 66.6% among women and 62.4% among men. Another affected group is childhood and adolescence: according to the 2016 School Census, overweight and obesity affect about 34% of the school population (3).

The country is in the process of implementing a tax reform that includes the review and modification of the value added tax (VAT). With the approval of the “*Ley de Fortalecimiento de las Finanzas Públicas”* (No. 9635) by the Legislative Branch in December 2018, different criteria have been established to tax foods in a differentiated manner. Products listed in the basic tax basket (*canasta básica tributaria* [CBT]) would be taxed at 1% VAT, while the rest of the products would be taxed at 13%. The products included in the CBT are defined by Presidential Decree and referred to the food products most consumed by the first quintile of the population according to the 2013 National Survey of Household Income and Expenditures (4). Subsequently, on 4 December 2020, Law No. 9914 called “*Definición de la Canasta Básica por el Bienestar Integral de las Familias“* was approved (5). The law establishes that the CBT will be constituted considering the products most consumed by the 30% of households with the lowest income, and “will value the inclusion of foods of high nutritional value, based on criteria such as the implementation of a balanced and diverse diet that meets the nutritional needs, culturally relevant and derived from the epidemiological profile of the population.”

This last point is fundamental given that international evidence indicates that lower-income households tend to opt for foods whose cost per calorie provided is lower, such as foods high in sugars, fats and sodium, and consume relatively fewer foods whose cost per calorie provided is higher, such as fruits and vegetables (6). As a consequence, the introduction of VAT and the definition of a CBT without nutritional criteria may widen the gap between the costs of foods of low nutritional value and foods of high nutritional value, negatively impacting access to nutritionally desirable foods for the entire population and, particularly, for the lower-income population.

This can have socially undesirable consequences. On the one hand, it can create a vicious circle of poor nutrition by reinforcing low-quality diets, especially among the most socially vulnerable population. On the other hand, it can generate changes in poverty profiles if the composition of the CBT is not taken into account. Finally, indirectly incentivizing unhealthy diets can increase the development of non-communicable diseases in the medium and long term (7–10, 12, 14). Then, it is necessary to elaborate basic tax baskets with nutritional criteria and reject the logic of elaborating baskets whose composition barely covers the nutritional minimums without considering the other components of the right to food. According to the recommendations of the Institute of Nutrition of Central America and Panama (INCAP) for the elaboration of basic food baskets, it is necessary to include locally produced foods with high nutritional content and reject the inclusion of industrialized foods with low nutritional content and high caloric content. In this context, the CBT should not be far from this reality.

In the case of Costa Rica, a 2015 study by Vargas and Elizondo analyzed the price elasticity^1^ of food demand, identifying two groups of foods: those whose demand is inelastic (quantity demanded reacts less than proportionally to price changes) and those whose demand is elastic (quantity demanded reacts more than proportionally to price changes). The findings suggest that the introduction of taxes on high-calorie, low-nutrient products, such as soft drinks and cookies, could significantly reduce the consumption of these foods.

Knowing the extent to which food demand reacts to price changes makes it possible to anticipate changes in the quantities demanded as a result of fiscal policy changes (e.g., introduction of excise taxes or changes in VAT). It also allows measuring potential substitution and complementary effects between food groups, and the nutritional effects of fiscal policies. It is particularly relevant for the case of Costa Rica, since the content of the CBT is currently under discussion, and would incorporate a nutritional component for the selection of food products.

In this study, we estimate income^2^ and price elasticities (uncompensated)^3^ of different food groups for Costa Rican households using the *Encuesta Nacional de Ingresos y Gastos de los Hogares 2018* (15) and using a Quadratic Almost Ideal Demand System (QUAIDS) model. Price elasticities estimated from demand system models such as QUAIDS are key elements to measure the impact of fiscal policies on household spending for specific food groups. The objective of this work is to generate technical and reliable information for fiscal policy decision making to promote actions to facilitate the consumption of healthier foods, especially in population with social vulnerability, and under the progressive approach of the current government. The results of this study could be used to redesign or evaluate current fiscal policies related to food and beverage consumption.

## Methodology

### Data Sources

The *Encuesta Nacional de Ingresos y Gastos de los Hogares 2018* (15) conducted by the National Institute of Statistics and Census (INEC) was used to estimate the model and the elasticity calculations. The cross-sectional survey collects household-level information on spending on different goods and services (expenditures and quantities), as well as socioeconomic and demographic information on 7,046 households. It is used to elaborate the Consumer Price Index, basic food baskets, to define poverty and perform other consumption/socioeconomic analyses. The survey is representative for 6 different regions covering Costa Rica. The information was collected between February 2018 and March 2019 for 36 weeks and over 10-day periods. For the purpose of this study, all food and beverage product records are taken into account with the exception of donations, obtaining a final sample of 6,972 households. Donations have been eliminated because they do not represent any purchase decision by the individual based on a certain price.

### Variables

Eleven food and beverage groups were used for this study. The food and beverage groups used in this study are based on the *Clasificación del Consumo Individual por Finalidades* (CCIF classification), because they correspond to large food categories that we are interested in analyzing. The CCIF is a classification of household consumption expenditures that national statistical offices have developed on their own, and have used in various analytical applications (1, 11). They are sufficiently large categories to obtain reliable price and income elasticities, and sufficiently disaggregated to observe complementarities and substitutions between groups according to potential price and/or income changes. CCIF groups are used rather than nutritional groups in order to give an economic balance representing an individual's consumption. Foods are already classified according to the CCIF classification in the ENIGH 2018 (15). Table 1 shows the list of categories with the respective codes.

**Table 1:** Descriptive statistics of consumption by CCIF category: households reporting expenditure>0 (6,972 households).

**Deciles**		**2 first deciles (1,661 obs.)**	**3 first deciles (2,433 obs.)**	**7 other deciles (4,539 obs.)**	**Total sample (6,972 obs.)**
Number of households		1,661	2,433	4,539	6,972
Average monthly expenditure		1.99 × 10^7^	2.14 × 10^7^	3.49 × 10^7^	3.02 × 10^7^
**CCIF category**	**CCIF code**	**Households reporting expenditure** **>** **0**			
Bread and cereals	0111	1,562	2,288	4,216	6,504
Meat	0112	1,175	1,730	3,252	4,982
Fish	0113	659	965	1,894	2,859
Milk, cheese, and eggs	0114	1,351	1,979	3,831	5,810
Oils and fats	0115	965	1,385	2,091	3,476
Fruits	0116	589	943	2,596	3,539
Pulses and vegetables	0117	1,351	1,986	3,665	5,651
Sugar, jam, honey, chocolate, and sweets	0118	1,192	1,700	2,779	4,479
Condiments	0119	1,016	1,443	2,437	3,880
Coffee, tea, and cocoa	0121	953	1,365	1,951	3,316
Mineral waters, soft drinks, fruit, and vegetable juices	0122	842	1,268	2,581	3,849

*Average expenditure per month is expressed in 2020 CRC (CRC 615.2 = EUR 1).*

*Source: Own elaboration with data from INEC (15). Weighted values*.

Food expenditure percentages were calculated for each household by summing the expenditures within each group and then dividing by the total expenditure for the twelve categories. Unit price values were calculated for each household as the ratio of expenditure to quantity for each group.

### Demand Analysis: QUAIDS and Elasticity Calculations

The objective of the study is to understand the sensitivity of consumers with different economic situations to price changes, producing elasticities that report this sensitivity. To do so, we estimate a Quadratic Almost Ideal Demand System (QUAIDS) using Stata v.16.1. This model allows us to assess the extent to which demand reacts to price and income variations, hence allowing to anticipate variations in quantities demanded as a result of potential changes (e.g., the introduction of taxes or changes in the VAT rate). More details about the model can be found in Annex 1.

### Endogeneity and Missing Values

A proportion of households do not report purchases of the food and beverage categories we considered. This may be due to different reasons such as: the household does not consume these types of products or simply did not do so in that reference period of the survey. In fact, the ENIGH was not conducted for the purpose of this analysis in particular. It is used to elaborate the Consumer Price Index, basic food baskets, to define the poverty line as well as other consumption/socioeconomic analyses. For this reason, depending on the decade considered, some households report consuming the different food categories and some others do not, but it is still useful because the reports balance each other out, and allow the aforementioned studies to be carried out.

Nevertheless, the ENIGH is the only source of information in Costa Rica with timely and current data on food quantities and expenditures. It has been proven that income and expenditure surveys are a tangible and reliable option to develop this type of analysis if the data is analyzed cautiously.

It is possible to implement a specific methodology to take into account this issue of missing values (biased parameter estimates). Cohorts can be created to group households and have complete information for a certain defined number of groups. This methodology has been used by Mendoza-Velázquez (13, 16). In our case, 200 groups were created from the income variable (net, current, per capita and without rental value) of the database. This method reduces data variability, but it gives elasticities that represent better the totality of households, and not only the households that report consuming more food categories, who in general have similar socio-demographic characteristics.

This aggregation of hlds also mitigates the endogeneity problems that naturally exist in these demand systems: the unit values are calculated using other variables of the system (expenditures and quantities per food category); hence they are endogenous. The consequence is that the obtained expenditure percentages may depend on other factors. The creation of 200 income groups should generate reliable average unit values for each group, smoothing out the variations that may exist in food quality as well.

## Results

Table 1 presents general sociodemographic descriptive data. Results will also be presented by deciles of net current income per capita (without rental value) for the first two deciles and first three deciles of the population. Results are presented in this way because the basic food basket in Costa Rica is usually based on the consumption patterns from the first two deciles of the population in terms of income.

Tables 1–3 show the percentage of households reporting consumption in each category, the average expenditure per food group and the average unit values in the different decile groups. The percentage of households reporting expenditure greater than zero allows us to identify the food categories most consumed by households, and to evaluate the problem of missing values.

**Table 2:** Descriptive statistics of consumption by CCIF category: average expenditure (6,972 households).

**Deciles**	**2 first deciles (1,661 obs.)**	**3 first deciles (2,433 obs.)**	**7 other deciles (4,539 obs.)**	**Total sample (6,972 obs.)**
**CCIF category**	**Average expenditure (% of total food expenditure)**			
Bread and cereals	24.88%	24.42%	20.38%	21.37%
Meat	16.76%	17.06%	17.80%	17.63%
Fish	4.06%	3.98%	4.76%	4.57%
Milk, cheese, and eggs	13.73%	14.0%	14.97%	14.69%
Oils and fats	4.12%	3.87%	2.75%	3.03%
Fruits	3.27%	3.81%	7.61%	6.68%
Pulses and vegetables	12.27%	12.54%	13.08%	12.99%
Sugar, jam, honey, chocolate, and sweets	6.73%	6.38%	4.85%	5.21%
Condiments	5.21%	4.98%	5.38%	5.26%
Coffee, tea, cocoa	4.85%	4.73%	3.26%	3.63%
Mineral waters, soft drinks, fruit, and vegetable juices	4.21%	4.21%	4.86%	4.70%

*Average expenditure per month is expressed in 2020 CRC (CRC 615.2 = EUR 1).*

*Source: Own elaboration with data from INEC (15). Weighted values*.

**Table 3:** Descriptive statistics of consumption by CCIF category: unit values (6,972 households).

**Deciles**	**2 first deciles (1,661 obs.)**	**3 first deciles (2,433 obs.)**	**7 other deciles (4,539 obs.)**	**Total sample (6,972 obs.)**
**CCIF category**	**Average unit values for 100 grams**			
Bread and cereals	157	161	223	201
Meat	295	298	367	343
Fish	547	556	618	597
Milk, cheese, and eggs	202	201	214	210
Oils and fats	147	152	206	184
Fruits	139	142	161	156
Pulses and vegetables	124	122	131	128
Sugar, jam, honey, chocolate, and sweets	230	242	421	353
Condiments	429	426	471	454
Coffee, tea, cocoa	458	472	654	579
Mineral waters, soft drinks, fruit, and vegetable juices	87	90	96	94

*Average expenditure per month is expressed in 2020 CRC (CRC 615.2 = EUR 1).*

*Source: Own elaboration with data from INEC (15). Weighted values*.

The category “Fish” has the lowest percentage of households reporting purchases, followed by “Coffee, tea and cocoa.” On the contrary, 93.2% of households report buying “Bread and cereals,” and 83.2% “Milk, cheese and eggs.” There is a pronounced difference in fruit consumption between the first two deciles of the population (35.5%) and the last seven (57.2%). “Fish” is the group with the highest average unit value per gram, followed by: “Coffee, tea and cocoa”; “Condiments”; “Sugar, jam, honey, chocolate and sweets” and “Meat.”

Table 4 is a summary of the key values, highlighting the problem of missing values with the column “Proportion of households with no consumption (percentage),” which is the percentage of households out of the total sample that do not report consuming a certain food category. For example, 59% of the total sample (4,113 households) do not report consuming fish and 6.71% (468 households) do not report consuming bread and cereals (see Table 1, households reporting expenditure > 0).

**Table 4:** Summary of descriptive statistics of consumption by CCIF category (6,972 households).

**CCIF category**	**Average expenditure (% of total food expenditure)**	**Average unit values for 100 grams**	**Proportion of households without consumption (%)**	**Number of missing values**
Bread and cereals	21.37%	201	6.71%	468
Meat	17.63%	343	28.54%	1,990
Fish	4.57%	597	59%	4,113
Milk, cheese, and eggs	14.69%	210	16.67%	1,162
Oils and fats	3.03%	184	50.21%	3,496
Fruits	6.68%	156	50.14%	3,443
Pulses and vegetables	12.99%	128	18.95%	1,321
Sugar, jam, honey, chocolate, and sweets	5.21%	353	35.76%	2,493
Condiments	5.26%	454	44.35%	3,092
Coffee, tea, cocoa	3.63%	579	52.44%	3,56
Mineral waters, soft drinks, fruit, and vegetable juices	4.70%	94	44.79%	3,123

*Average expenditure per month is expressed in 2020 CRC (CRC 615.2 = EUR 1).*

*Source: Own elaboration with data from INEC (15). Weighted values*.

Tables 5, 6 present the income and uncompensated price elasticities (own and cross-price elasticities) of the QUAIDS model. To facilitate the interpretation of the following tables, the different types of elasticities obtained are defined and explained:

- **Income elasticity:** reveals how much the quantity demanded for a good or food (or food category) varies as a percentage against percentage changes in consumers' income levels.
- **Price elasticity:** reveals how much the quantity demanded for a good or food (or food category) varies as a percentage against percentage changes in its price. There are two types of price elasticities:

- **Uncompensated (own) price elasticity:** takes into account the influence of prices and income on utility maximization.
- **Uncompensated (cross-price) price elasticity:** reveals the change in the quantity demanded for a good or food (or food category) when the price of another good, product or food category changes. This elasticity reveals whether two goods or groups of goods are complementary or substitutes.

**Table 5:** Income elasticities from QUAIDS model (6,972 households).

**CCIF category**	**Mean**	**Standard**	**Min**	**Max**
		**deviation**		
Bread and cereals	0.99	0.002	0.98	0.99
Meat	1.00	0.001	1.00	1.00
Fish	0.97	0.012	0.91	0.99
Milk, cheese, and eggs	1.01	0.002	1.01	1.02
Oils and fats	1.01	0.005	1.00	1.06
Fruits	1.09	0.063	1.02	1.59
Pulses and vegetables	1.01	0.002	1.01	1.02
Sugar, jam, honey, chocolate, and sweets	0.96	0.011	0.92	0.98
Condiments	0.96	0.017	0.86	0.99
Coffee, tea, cocoa	0.92	0.033	0.73	0.97
Mineral waters, soft drinks, fruit and vegetable juices	1.07	0.021	1.03	1.16

*Own elaboration with data from INEC (15). Weighted values*.

**Table 6:** Average expenditures, unit values, share of zero consumption and elasticities.

**CCIF category**	**Average expenditure (% of total food expenditure)**	**Average unit values for 100 grams**	**Proportion of households without consumption (%)**	**Income elasticities**	**Uncompensated own price elasticities**
Bread and cereals	21.37%	201	6.71%	0.99	−1.19
Meat	17.63%	343	28.54%	1.00	−1.00
Fish	4.57%	597	59%	0.97	−0.96
Milk, cheese and eggs	14.69%	210	16.67%	1.01	−1.03
Oils and fats	3.03%	184	50.21%	1.01	−0.70
Fruits	6.68%	156	50.14%	1.09	−0.99
Pulses and vegetables	12.99%	128	18.95%	1.01	−1.00
Sugar, jam, honey, chocolate, and sweets	5.21%	353	35.76%	0.96	−0.89
Condiments	5.26%	454	44.35%	0.96	−0.41
Coffee, tea, cocoa	3.63%	579	52.44%	0.92	−0.39
Mineral waters, soft drinks, fruit, and vegetable juices	4.70%	94	44.79%	1.07	−1.14

*Average expenditure per month is expressed in 2020 CRC (CRC 615.2 = EUR 1).*

*Source: Own elaboration with data from INEC (15). Weighted values*.

Uncompensated elasticities were chosen because they take into account the influence of prices and income in the maximization of utility, the compensated ones only prices. Annex 2 presents the uncompensated price elasticities. The main observations on Table 5 and Annex 2 are the following:

### Income Elasticities (Table 5)

All elasticities have positive values, and most of them are very close to one (Table 5). It means that the food categories correspond to foods that are normal goods (elasticities between zero and one). When income increases, consumption of normal goods increases almost proportionally to the increase in income. For two categories, the elasticities are slightly higher than one: “Fruits” (1.09) and “Mineral waters, soft drinks and juices” (1.07). For interpretation purposes, income elasticities higher than one correspond to “luxury” categories/foods. It means that when income increases, consumption increases more than the increase in income. For the category “Fruits,” it means that when income increases by 10 percent, consumption increases by 10.9 percent. This is the case when there is perfect price transmission. In reality, it is rarely verified since market structure, education, households culinary skills or even time use for example, also play an important but often unobservable role in consumption patterns. Normally, meat, fish and sugary products are expected to be “luxury” categories.

Income elasticities provide information on the importance of income in consumption patterns according to food categories. In this case, no essential differences are observed between food categories. It is essential to take this result into account when we want to measure the potential effect of fiscal policy changes. This is also why uncompensated price elasticities are more suitable for drawing conclusions. They allow us to take into account the influence of prices and income on the maximization of utility, while the compensated ones only take prices into account.

### Own Price Elasticities (Annex 2)

The values on the diagonal of Annex 2 represent the own uncompensated price elasticities, while the values not on the diagonal represent the cross uncompensated price elasticities (see next section).

All elasticities are negative. A negative price elasticity means that when the price goes up, consumption decreases, i.e., there is an inverse price-quantity relationship. The most elastic categories (elasticities far from zero) are the following: “Bread and cereals” (−1.19); “Mineral waters, soft drinks and fruit and vegetable juices” (−1.14); “Milk, cheese and eggs” (−1.03); “Pulses and vegetables” (−1.00) and “Meat” (−1.00). In theory, an elastic food is a food whose variation in consumption is higher than the observed price variation, i.e., the quantity demanded reacts more than proportionally to price changes. This is the case for the category “Mineral waters, soft drinks and fruit and vegetable juices.” The result means that when the price of the category rises by 10%, consumption decreases by 11.4%. A VAT of 13% on this food category gives for example a reduction in consumption of 14.8%. If the elasticities are equal to one, it means that the quantity demanded reacts proportionally to price changes, this is the case for the mentioned categories (“Meat” and “Vegetables”). For the rest of the categories, the elasticities are inelastic, especially for the following categories: “Coffee, tea and cocoa” (−0.39); “Condiments” (−0.41), and in a second tier “Oils and fats” (−0.70).

Table 6 summarizes the main results of the study (Tables 4–6).

### Cross Price Elasticities

Cross-price elasticities allow to identify complementarities and substitutions between groups. Table 7 shows the foods with the highest substitution ratio, i.e., substitute groups (elasticities> 0). A substitute good is a good capable of satisfying the same need as another good. When the price of such a good increases, the demand for one of its substitutes increases. A complementary good is a good which consumption level is linked to the price of another good. When the price of such a good increases, the demand for one of its complements decreases.

**Table 7:** Main substitutions between groups.

**Change in price/change in quantity**	**Uncompensated cross price elasticities**
Bread and cereals/fruits	0.47
Fruits/oils and fats	0.36
Milk, cheese and eggs/oils and fats	0.31
Meat/fish	0.26

*Other groups are substitutes but the table presents the strongest relationships.*

*Source: Own elaboration with data from INEC (15)*.

The results show that an increase in the price of bread and cereals contributes to an increase in fruit consumption. Similarly, an increase in the price of fruits contributes to an increase in the consumption of oils and fats; and of milk, cheese and dairy products to an increase in the consumption of oils and fats. Also, an increase in the price of meat contributes to an increase in fish consumption. In these cases, the increase in consumption is quite small (<10%). For instance, a 10% price increase of the category “Bread and cereals” would lead to an increase in the fruits quantities consumed by 4.7%. It is important to take these substitutions into account because these results show that changing the price of one food category changes the consumption of others.

With respect to the complementary goods, if the prices of the “Bread and cereals” group rise, there is on the one hand a reduction in its consumption (−1.19), and on the other hand to reduce the consumption of other food groups such as “Oils and fats” (−0.69) and “Sugar, jam, honey, chocolates and sugar confectionery” (−0.29). Indeed, the consumption of bread is usually associated to some products of these two categories. On the other hand, the consumption of fruits (0.47), fish (0.23) and milk, cheese and eggs (0.21) would increase. In general terms, these would appear to be nutritionally healthy variations. It is worth noticing that, in the event of a rise in the price of fruit, there will be a drop in its consumption (−0.99), but consumption of pulses and vegetables (−0.15) and fish (−0.32) would also slightly drop, while consumption of sugar (0.13) and oils and fats (0.32) would slightly rise. All these variations would not be very healthy in the evolution of the diet.

The price increase in the sugar group, in addition to reducing the consumption of these products, would contribute to a decrease in the consumption of oils and fats and an increase in the consumption of fruits. Finally, it is important to mention that a rise in the price of meat would lead to an increase in fish consumption by substitution (0.26). Therefore, if greater fish consumption were to be encouraged, the inclusion of a smaller number of meat products in the CBT could be an alternative to be considered. The increase in the price of meat would also lead to a reduction in the consumption of soft drinks and juices (−0.27).

## Discussion

The results are consistent with the results found by other studies in Latin America, especially for the following categories: “Mineral waters, soft drinks, fruit and vegetable juices”; “Fruits”; “Milk, cheese and eggs”; “Meat”; “Sugar, jam, honey, chocolate and sweets” and “Fish.” For the category “Bread and cereals,” we found a more elastic result than the literature. Variations in the estimates may be given by the specifications of the models (AIDS or QUAIDS), the food groups elected and/or by the data used (sample size, chosen demographic variables).

Comparing the results with the Vargas and Elizondo (17) study in particular, we have similar results except for the categories “Meat” and “Fish.” In their study, the results are less elastic. The Vargas and Elizondo study also calculates elasticities based on the ENIGH in Costa Rica, but does not use a QUAIDS model and presents different food groups than those presented in this study. This may explain the differences obtained. Meat and fish are comparatively expensive foods, and this is why we find more elastic price elasticities. Similarly, the categories “Milk, cheese and eggs” and “Vegetables” are more elastic in our study. It means that the consumption of vegetables can be encouraged with a decrease in their price. On the contrary, the category “Sugar, jam, honey, chocolate and sweets” is less elastic in this study. This information confirms that in order to discourage the consumption of this type of food, a more complete nutritional strategy should be articulated, including public campaigns, advertising bans, education, among others. Besides, an additional increase in the taxation of unhealthy products such as soft drinks would reduce the current high consumption rates. In this study and others, soft drinks have an elasticity greater than one, which means the effect of an increase in price on consumption would be significant. The more price-elastic a food is, the more efficient it is, for the reduction of its consumption, to increase its price (taxes, VAT increase, etc).

Table 8 presents the results obtained in similar studies. When the categories were different, the results of the studies were averaged to correspond to the groups used in this study. Not all studies look at all the categories incorporated in this paper.

**Table 8:** Uncompensated price elasticities.

**CCIF category**	**Present study (2020)**	**Vargas and Elizondo (17)**	**Caro et al. (18)**	**Nimanthika Lokuge et al. (19)**	**Mendoza-Velázquez (16)**	**Caro et al. (20)**
**Country**	**Costa Rica**	**Costa Rica**	**Colombia**	**Sri Lanka**	**Mexico**	**Chile**
Bread and cereals	−1.19	−1.00	−0.85	−0.67	−0.46	−0.67
Meat	−1.00	−0.65	−0.84	−1.30	−0.49	−1.13
Fish	−0.96	−0.30		−0.98		−1.10
Milk, cheese and eggs	−1.03	−0.85	−0.94	−0.98		
Oils and fats	−0.70	−0.95			−0.58	
Fruits	−0.99	−0.80	−0.96	−0.80		
Pulses and vegetables	−1.00	−0.70	−0.96	−0.80	−0.7	
Sugar, jam, honey, chocolate and sweets	−0.89	−1.00	−0.80			−0.80
Condiments	−0.41		−1.01			
Coffee, tea, cocoa	−0.39	−0.50	−1.35			−1.37
Mineral waters, soft drinks, fruit and vegetable juices	−1.14	−0.10	−1.62			−1.00

*Elasticities are significant at p < 0.05.*

*Source: Own elaboration with data from INEC (15)*.

We identified that there was substitution between the “Fruits” category and two other categories: “Oils and fats” and “Bread and cereals.” For example, if one wants to increase the consumption of fruits, one should keep their price low so as not to direct consumption toward more foods of the “Oils and fats” category, as well as raise the price of bread and cereals. This result makes sense because the unit values of fruits and oils and fats are quite similar, also with the unit value of pulses and vegetables. Nevertheless, it can be intuited that consumption is shifted toward more oils and fats because they are more convenient foods to eat and cook with (current lifestyles). There is also a preference to consume foods from the category “Bread and cereals” when the price of fruits goes up because these foods are preferred and consumed a lot in Costa Rica. There is also substitution between the “Milk, cheese and eggs” category and the “Oils and fats” category. For example, if we wanted to increase the consumption of milk, cheese and eggs, we would have to keep their prices sufficiently low. The tendency is to substitute these more expensive fats with cheaper fats of the “Oils and fats” category.

Substitution effects between food categories are important elements when constructing a CBT with nutritional criteria, as they determine whether the CBT can ultimately have positive effects on health. For the CBT it would mean, for instance, that to promote fruit consumption and avoid shifting consumption to more oils and fats, limiting the inclusion of products from the categories “Bread and cereals” in the basket may be an option. It is necessary to choose carefully the products from this category that will be part of the basket, based on nutritional and/or food security criteria. On the contrary, more fruits should be included in the CBT for positive health effects. As suggested by the analysis, a rise in the price of “Bread and cereals” also leads to a decrease in oils, fats and sweets consumption, which induces a general positive impact on health.

## Conclusion

Nutritional visions in the definition of any fiscal measure involving food products is vital, due to the proven impact that the variation of their price has on food choices and therefore on the health of the population. The inclusion or removal of products from the CBT has nutritional and public health effects, and it is essential that these are taken into consideration when selecting the CBT. It is important to encourage the consumption of particularly healthy food groups such as fruits, pluses and vegetables to promote healthier diets. The consumption of fruits, pulses and vegetables can be significantly encouraged through price reductions and, for example, reduced VAT. A greater number of these types of products in the CBT will be an indicator of its healthy character. It is important at the same time to discourage the consumption of particularly unhealthy food groups such as sugar-sweetened beverages and the group of sugar, jam, honey, chocolate and sweets with fiscal measures (VAT increase and/or excise taxes), but also with other complementary measures (public campaigns, banning of advertisements, education, among others), since we observed the consumption of some of these products was not strongly elastic to price changes.

Finally, it is important that in each food group, the consumption of the healthiest foods within each group be fiscally promoted. Being more nutritionally selective when choosing products to be included in the basic food basket is essential due to the negative impact some food products can have on the overall diet. In this regard, effects of substitution and complementarity between food groups must be taken into account. Substitutions and complementarity with the food group “Bread and cereals” is a case in point in this study.

## Data Availability Statement

The original contributions presented in the study are included in the article/supplementary materials, further inquiries can be directed to the corresponding author.

## Author Contributions

RR and CM: conceptualization, validation, supervision, and project administration. RR, CM, and ED: methodology. ED, RR, and MM: software and data curation. ED and RR: formal analysis and investigation. CM: resources and funding acquisition. ED: writing–original draft and visualization. ED, CM, and RR: writing—review and editing. All authors contributed to the article and approved the submitted version.

## Funding

This work was supported by granting agency: FAO Grant Number: tbc.

## Conflict of Interest

The authors declare that the research was conducted in the absence of any commercial or financial relationships that could be construed as a potential conflict of interest.

## Publisher's Note

All claims expressed in this article are solely those of the authors and do not necessarily represent those of their affiliated organizations, or those of the publisher, the editors and the reviewers. Any product that may be evaluated in this article, or claim that may be made by its manufacturer, is not guaranteed or endorsed by the publisher.

## Footnotes

^1^The price elasticity of demand reveals how much the quantity demanded for a good or food (or category of food) varies with changes in its price.

^2^Income elasticity of demand reveals how much the quantity demanded for a good or food (or food category) varies with changes in consumers' income levels.

^3^Uncompensated price elasticity takes into account the influence of prices and income on utility maximization, compensated price elasticity only prices.
