# Supplementary material for: Fiscal Reform in Costa Rica: Price Elasticities of Major Food Categories to Inform Decision-Making
Source: Front Nutr. 2022 Apr 27;9:836501. doi: 10.3389/fnut.2022.836501 (PMC9093704; doi:10.3389/fnut.2022.836501)
Supplement: Supplementary file 1 [file Data_Sheet_1.pdf]

Annex 1. QUAIDS model

The model is as follows (Banks *et al.*, 1997):

$$w_i = \alpha_i + \sum_{j \in I} \gamma_{ij} \ln p_j + \beta_i \ln\left(\frac{m}{a(p)}\right) + \frac{\lambda_i}{b(p)}\left(\ln\left(\frac{m}{a(p)}\right)\right)^2 \ \forall\, i \in I$$

$$\ln a(p) = \alpha_0 + \sum_{j \in I} \alpha_j \ln p_j + \frac{1}{2}\sum_{l \in I}\sum_{j \in I} \gamma_{lj} \ln p_l \ln p_j$$

$$b(p) = \prod_{j \in I} p_j^{\beta_j}$$

Where:

w_i y p_i are the expenditure and price percentages for food category i;

m is the total food expenditure per household;

I represents all the food categories (in this case 11);

α_i , γ_{ij}, β_i y λ_i are the parameters to be estimated.

According to Deaton and Muellbauer (1980), linear restrictions on the parameters are necessary for the model to correspond to the economic theory of demand. They are the following:

$$\textstyle\sum_{i \in I} \alpha_i = 1,\ \sum_{i \in I} \beta_i = 0,\ \sum_{i \in I} \lambda_i = 0,\ \sum_{i \in I} \gamma_{ij} = 0\ \ \forall\, i \in I$$

$$\textstyle\sum_{j \in I} \gamma_{ij} = 0\ \ \forall\, i \in I,\ \gamma_{ij} = \gamma_{ji}\ \forall\, i,j \in I \text{ (symmetry}^{1}\text{)}$$

$$\textstyle\sum_{i \in I} \rho_{ik} = 0\ \ \forall\, k \in K \text{ (zero degree homogeneity on prices}^{2}\text{)}$$

Finally, the demand system must be equal to 1, since the expenditure shares must add up to 100 percent (these are percentages of expenditure over total expenditure):

$$\sum_{i \in I} w_i = 1$$

[1] The substitution or complementarity effects between food categories are symmetrical (direction and magnitude).
[2] If prices and income change in the same way, quantities demanded are not affected.

Total food expenditure, being highly correlated to income, is used in the model to calculate income elasticities. The elasticities (income and price) were calculated for the mean values of the variables. They can be estimated from the following equations (μ_iy μ_{ij}):

$$\mu_i = \frac{\partial w_i}{\partial \ln m} = \beta_i + \frac{2\lambda_i}{b(p)} \ln\left(\frac{m}{a(p)}\right)$$

$$\mu_{ij} = \frac{\partial w_i}{\partial \ln p_j} = \gamma_{ij} - \mu_i \left(\alpha_j + \sum_k \gamma_{jk} \ln p_k\right) - \frac{\lambda_i \beta_j}{b(p)} \left(\ln\left(\frac{m}{a(p)}\right)\right)^2$$

Income elasticities can be obtained from the following expression:

$$\varepsilon_i = \frac{\mu_i}{w_i} + 1$$

The uncompensated price elasticities are given by the following equation:

$$\varepsilon_{ij}^u = \frac{\mu_{ij}}{w_i} - \delta_{ij}$$

Other methods commonly used are Almost Ideal Demand System (AIDS) models. The advantage of QUAIDS is that, unlike AIDS, it allows greater flexibility in the income and expenditure curves (Engel curves). It means that it allows the inclusion of a non-linear expenditure function.

We assumed weak budget separability, therefore modelling the total demand for food at home, divided in eleven groups. We used the expansion factors in order to take into account the survey design in our estimates.
