# Supplementary material for: Fiscal Reform in Costa Rica: Price Elasticities of Major Food Categories to Inform Decision-Making
Source: Front Nutr. 2022 Apr 27;9:836501. doi: 10.3389/fnut.2022.836501 (PMC9093704; doi:10.3389/fnut.2022.836501)
Supplement: Supplementary file 2 [file Data_Sheet_2.docx]

# Annex 2. Uncompensated price elasticities from the QUAIDS model (6 972 households)

| Change in quantity | Change in price | | | | | | | | | | |
| --- | --- | --- | --- | --- | --- | --- | --- | --- | --- | --- | --- |
|  | **111** | **112** | **113** | **114** | **115** | **116** | **117** | **118** | **119** | **121** | **122** |
| 111 | -1.1878722 | .07627355 | .04708748 | .13859468 | -.09955099 | .1337758 | -.02400962 | -.07027901 | -.00694228 | -.03999459 | .04634192 |
| 112 | .09322565 | -.99994276 | .06476562 | -.13698653 | .01141682 | -.01238999 | .05895604 | -.02532486 | .02278568 | -.0067876 | -.06698431 |
| 113 | .22851064 | .2584827 | -.96299248 | -.27791837 | .04832681 | -.32175703 | .00955154 | .05467815 | -.02392643 | .13366723 | -.11953102 |
| 114 | .20748456 | -.16621397 | -.08437272 | -1.0265419 | .06522687 | .02504299 | .02395997 | .05102094 | -.07421837 | -.06522345 | .03190351 |
| 115 | -.69440555 | .06500356 | .06920808 | .30609847 | -.70481225 | .362719 | .12063956 | -.30426408 | -.19383522 | -.08049022 | .04590794 |
| 116 | .46542351 | -.03621565 | -.22932589 | .06174753 | .18062742 | -.98897858 | -.31320211 | .11477697 | -.16228658 | -.17558979 | .01244048 |
| 117 | -.04080109 | .08045028 | .0027774 | .02805348 | .02918873 | -.15073681 | -1.000235 | .02234143 | -.06825009 | .06745814 | .0180704 |
| 118 | -.28592963 | -.0833606 | .04663341 | .13740433 | -.17787481 | .13307626 | .05459323 | -.89055765 | .09084706 | -.06715348 | .07515819 |
| 119 | -.03072146 | .07747932 | -.02005572 | -.20798006 | -.11528919 | -.19248388 | -.16663543 | .09097873 | -.41113043 | .0207857 | -.00734187 |
| 121 | -.23606634 | -.03133475 | .16010972 | -.25818156 | -.06768546 | -.2935999 | .23690898 | -.09833821 | .02970481 | -.39224743 | .01784429 |
| 122 | .15529041 | -.26676074 | -.14127637 | .14074497 | .03677334 | .11202594 | .08080266 | .04990058 | -.05189906 | -.04049113 | -1.1373773 |

Source: Own elaboration with data from INEC (2018).
